# Estimated Incidence of Electric Scooter Injuries in the US From 2014 to 2019

**DOI:** 10.1001/jamanetworkopen.2020.14500

**Published:** 2020-08-31

**Authors:** Kevin Xavier Farley, Matthew Aizpuru, Jacob M. Wilson, Charles A. Daly, John Xerogeanes, Michael B. Gottschalk, Eric R. Wagner

**Affiliations:** 1 Department of Orthopaedic Surgery, Emory University School of Medicine, Atlanta, Georgia; 2 Department of Surgery, Mayo Clinic, Rochester, Minnesota

## Abstract

This cross-sectional study uses data from the US Consumer Product Safety Commission’s National Electronic Injury Surveillance System to assess trends in the prevalence of electric scooter injuries in the US from 2014 to 2019.

## Introduction

In late 2017, scooter-share companies began distributing electric scooters (e-scooters) in major cities, leading to an increase in their use.^1,2,3^ Data from the 2019 United States Consumer Product Safety Commission’s National Electronic Injury Surveillance System (NEISS) have recently become available, allowing continued analysis of nationwide trends in e-scooter injuries since the widespread expansion of scooter-share services. The purpose of this cross-sectional study was to assess the incidence of and trends among e-scooter injuries in the US from 2014 to 2019.

## Methods

This cross-sectional study used data from the NEISS, a statistically valid surveillance system designed to collect data on patients seen in emergency departments (EDs) with injuries related to consumer products by creating a probability sample from approximately 100 hospitals in the US. The NEISS has previously been used to examine e-scooter injuries.^1,2^ It was queried for cases with product code 5042 (scooters/skateboards, powered) and identified 5171 unweighted cases. Electric-scooter injuries were then isolated from the sample through a search with inclusion of the term *scooter* and exclusion of the terms *hover*, *board*, *skate*, *wheelchair*, *motorbike*, and *motorcycle*, leaving 1823 cases available for analysis. The Emory University institutional review board waived the requirement for review because the study did not meet the definition of human subjects research. This study followed the Strengthening the Reporting of Observational Studies in Epidemiology (STROBE) reporting guideline.

Weighted estimates and 95% CIs were created within the complex samples function of SPSS Statistics, version 26.0 (IBM). Data from the US Census Bureau were used to create weighted incidences per 100 000 population. To evaluate trends from 2014 to 2019, regression with a log function was used while accounting for the standard error of the estimates. The significance level was set at *P* < .05 using a 2-sided test.

## Results

There were an estimated 70 644 (95% CI, 53 838-87 448) ED visits for e-scooter–related injuries from 2014 to 2019. The mean (SD) age of those injured was 31.3 (21.24) years (95% CI, 29.4-33.2 years), and 63.9% were men. The estimated number of ED visits for e-scooter injuries increased from 4881 (95% CI, 4086-5676) in 2014 to 29 628 (95% CI, 14 919-44 338) in 2019, with an increase from 8269 visits (95% CI, 5409-11 130 visits) in 2017 to 15 522 visits (95% CI, 8280-22 763 visits) in 2018. The population-adjusted incidence increased from 1.53 per 100 000 capita (95% CI, 1.28-1.78 per 100 000 capita) in 2014 to 9.22 per 100 000 capita (95% CI, 4.64-13.79 per 100 000 capita) in 2019 (Figure). Incident ED visits for e-scooter–related injuries increased most substantially among individuals aged 15 to 24 years and 25 to 39 years (Figure, Table).

**Figure.**
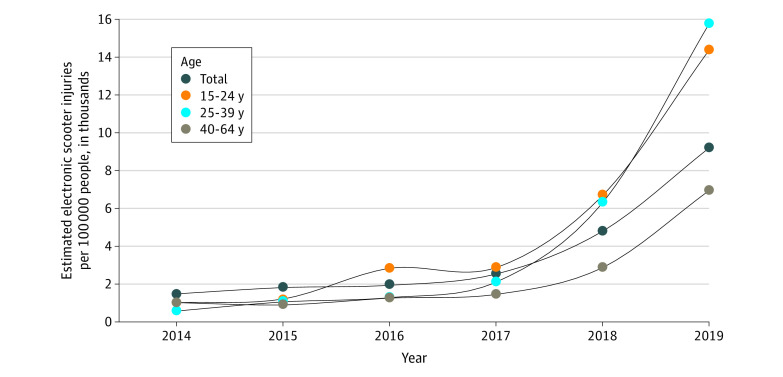
Trends in Weighted Incidence of Electric Scooter Injuries Seen in Emergency Departments in the US From 2014 to 2019 by Select Age Groups

**Table.** Patient Demographic and Injury Characteristics Associated With Electric Scooter Injuries Seen in Emergency Departments in the US From 2014 to 2019

Characteristic	Year	Weighted estimate (95% CI)^a^	Incidence, per 100 000 population (95% CI)	Count	Change, %	*P* value^a^
Age group						
Total	2014	4881 (4086-5676)	1.53 (1.28-1.78)	124	507	.009
	2015	5977 (4545-7409)	1.86 (1.41-2.31)	135		
	2016	6366 (4716-8016)	1.98 (1.47-2.49)	164		
	2017	8270 (5409-11 130)	2.57 (1.68-3.46)	201		
	2018	15 522 (8280-22 763)	4.83 (2.58-7.08)	397		
	2019	29 628 (14 919-44 338)	9.22 (4.64-13.79)	803		
≤14 y	2014	2265 (1392-3139)	3.55 (2.18-4.92)	67	63	.12
	2019	3699 (2344-5054)	5.89 (3.73-8.05)	128		
15-24 y	2014	453 (0-926)	1.03 (0.00-2.11)	10	1271	.001
	2015	542 (207-878)	1.23 (0.47-1.99)	17		
	2016	1253 (503-2002)	2.85 (1.15-4.56)	34		
	2017	1256 (263-2250)	2.89 (0.61-5.18)	27		
	2018	2896 (1242-4549)	6.71 (2.88-10.54)	84		
	2019	6217 (2890-9544)	14.40 (6.69-22.11)	176		
25-39 y	2014	392 (131-652)	0.62 (0.21-1.03)	14	2594	.002
	2015	708 (64-1351)	1.10 (0.10-2.10)	11		
	2016	865 (468-1263)	1.33 (0.72-1.93)	20		
	2017	1423 (419-2428)	2.15 (0.63-3.66)	30		
	2018	4241 (987-7495)	6.34 (1.47-11.20)	89		
	2019	10 555 (3434-17 676)	15.77 (5.13-26.42)	275		
40-64 y	2014	1088 (638-1539)	1.04 (0.61-1.48)	20	563	.01
	2015	968 (502-1435)	0.93 (0.48-1.38)	23		
	2016	1335 (571-2098)	1.28 (0.55-2.02)	23		
	2017	1539 (535-2544)	1.48 (0.51-2.44)	25		
	2018	3001 (691-5311)	2.89 (0.67-5.12)	61		
	2019	7211 (2869-11 552)	6.95 (2.76-11.13)	181		
≥65 y	2014	682 (171-1193)	1.47 (0.37-2.58)	13	185	.046
	2019	1946 (1012-2880)	3.71 (1.93-5.49)	43		
Sex						
Men	2014	2655 (1950-3360)	1.69 (1.24-2.14	70	635	.007
	2019	19 527 (8839-30 215)	12.12 (5.49-18.75	522		
Women	2014	2226 (1459-2992)	1.37 (0.90-1.85)	54	354	.02
	2019	10 101 (5512-14 691)	6.08 (3.32-8.85)	281		
Injury						
Traumatic brain injury^b^	2014	707 (315-1098)	0.22 (0.10-0.34)	21	503	.004
	2019	4262 (2113-6410)	1.33 (0.66-1.99)	118		
Fracture	2014	1247 (829-1665)	0.39 (0.26-0.52)	27	551	.02
	2019	8113 (3115-13 112)	2.52 (0.97-4.08)	233		
Disposition, admitted or transferred	2014	150 (41-258)	0.05 (0.01-0.08)	10	2039	<.001
	2019	3198 (1364-5031)	0.99 (0.42-1.57)	99		
Substance use						
Any	2019	2656 (494-4818)	0.83 (0.15-1.50)	73	NA	NA
Alcohol	2019	2340 (378-4302)	0.72 (0.12-1.31)	63		
Other drugs	2019	332 (50-614)	0.10 (0.02-0.19)	11		

Abbreviation: NA, not applicable.

^a^ *P* < .05 indicates a statistically significant change from 2014 to 2019.

^b^ Criteria for a traumatic brain injury included head injury with a concomitant diagnosis of concussion, internal organ injury, fracture, anoxia, or hemorrhage.

The head was the most common site of injury (27.1% of all injuries). Approximately 50% of head injuries included diagnoses that suggested a traumatic brain injury (a head injury with a concomitant diagnosis of a concussion, internal organ injury, fracture, anoxia, or hemorrhage), constituting 14.5% of the total injury pool. Of patients presenting with a potential traumatic brain injury, 17.4% were admitted to the hospital compared with 7.7% of patients without this diagnosis (*P* < .001). In 2019, an estimated 2656 (95% CI, 494-4818) e-scooter injuries involved substance use, with 88.1% involving alcohol (Table).

## Discussion

The estimated incidence of e-scooter injuries treated in EDs in the US nearly doubled between 2018 and 2019 despite various regulatory efforts and evidence highlighting this issue.^1,2^ Head injuries were the most common cause of visits to the ED, and traumatic brain injuries were prevalent among those injured. These results are troubling given that helmets are used by a minority of riders, helmet requirements have been eliminated in some areas, and riders often misunderstand road traffic laws that guide e-scooter use.^1,4,5,6^

Limitations of this study include possible underestimation of injuries, a lack of data outside ED visits, and the absence of information on helmet use. Estimates with fewer than 1200 weighted cases may be unstable. Furthermore, the weighted estimates presented here may not represent the true national incidence of scooter injuries seen in the ED, because scooters are unlikely to be equally distributed across sampled areas. Strengths include the use of a nationally representative data set.

The estimated incidence of e-scooter–related injuries seen in EDs increased between 2014 and 2019; thus, this appears to be an important public health issue. Continued efforts should be made to investigate strategies, such as required helmet use, enforcement of laws against riding under the influence of alcohol or drugs, and e-scooter safety education, to potentially mitigate the most serious injuries and keep riders safe.
